# On the Theory and Practice of Midwifery

**Published:** 1843-09-02

**Authors:** 


*On the Theory and Practice of Midwifery.* By Fleetwood Churchill, M. D., M. R. I. A., &c. &c. &c. *With Notes and Additions.* By Robert M. Huston, M. D. Professor of Materia Medica and General Therapeutics in the Jefferson Medical College of Philadelphia, etc. etc. etc. *With One Hundred and Sixteen Illustrations from Drawings by Bagg and others. Engraved by Gilbert.* Philadelphia: Lea & Blanchard. 1843. 8vo. pp. 519.

Dr. Churchill in his Preface states, that the object of the present publication "is to offer to the student of Midwifery a work, embracing the modern discoveries in the physiology of the uterine system, with all the recent improvements in practice, in a condensed form, amply illustrated, and at a moderate price."—(p. ix.)

The work amply fulfils, we think, all these indications. The physiological portion is more complete than that of any preceding treatise, English or French. We have some inaccuracies, but on the whole the actual condition of the science is fairly represented. This part we think misplaced. Midwifery authors, as well as teachers, are perpetually trespassing on the domain of the physiologist. Strictly, their duties are purely practical, and they but too often curtail their usefulness by indulging in idle speculations on generation, menstruation, &c. Our author, however, is not amenable to this charge, for the practice of obstetrics is ably treated, and embodies most, if not all the recent observations and suggestions.

The author regrets the absence of fuller references than his limits permitted. On that head we do not quite agree with him. However satisfactory they may be to the physician, or gratifying to an author's vanity, they only perplex the ordinary student, who is content with a well arranged summary, and clear digest of the best current opinions of the day on any given subject, as he has neither time nor, in a vast majority of cases, inclination to go further. In this respect we consider the present work much superior to our author's "Diseases of Females," which is encumbered by useless citations, and there is much occasional confusion in the text, from the "multitude of counsellors;" which, to our thinking, is often anything but wisdom.

The additions of Professor Huston are opportune in supplying accidental omissions, and in some instances deficiencies in the text. The remarks of the Editor on the Use of the Forceps are singularly judicious and apt. In Great Britain, the vast majority of practitioners of midwifery limit the use of the forceps to those cases in which the head has already passed through the superior strait, and has descended fully into the cavity of the pelvis. The result of this rule is seen in the appalling number of craniotomy cases. The tables recently published by Dr. Robert Lee in his "Clinical Midwifery," and in his Lectures in the Medical Gazette, show the lamentable frequency of this horrible operation amongst British practitioners. In Germany and in France the forceps are more constantly used, and with the happiest effects. We refer our readers, however, to the chapter itself, as one from which much useful information may be derived.

The work is beautifully printed, and illustrated in the best style of Gilbert with one hundred and sixteen cuts.

